# Combination adipose-derived mesenchymal stem cells-demineralized dentin matrix increase bone marker expression in periodontitis rats

**DOI:** 10.1016/j.sdentj.2023.07.019

**Published:** 2023-08-02

**Authors:** Desi Sandra Sari, Millenieo Martin, Ernie Maduratna, Hari Basuki Notobroto, Ferdiansyah Mahyudin, Ketut Sudiana, Nora Ertanti, Aristika Dinaryanti, Fedik Abdul Rantam

**Affiliations:** a Department of Periodontics, Faculty of Dentistry, Universitas Jember, Jember 68121, Indonesia; b Graduated Student, Faculty of Dentistry, Universitas Jember, Jember 68121, Indonesia; c Department of Periodontics, Faculty of Dentistry Universitas Airlangga, Surabaya 60132, Indonesia; d Department of Biostatistics and Demography, Faculty of Public Health, Universitas Airlangga, Surabaya 60132, Indonesia; e Department of Orthopaedic & Traumatology, Dr Soetomo General Hospital, Surabaya 60132, Indonesia; f Department of Pathology Anatomy, Faculty of Medicine, Universitas Airlangga, Surabaya 60132, Indonesia; g Stem Cells Research and Development Center, Universitas Airlangga, Surabaya 60132, Indonesia; h Department of Virology, Microbiology, and Immunology, Faculty of Veterinary Medicine, Universitas Airlangga, Surabaya 60132, Indonesia

**Keywords:** Adipose-Derived Mesenchymal Stem Cells, Demineralized Dentin Matrix, Regeneration Periodontal, Scaffold, Alveolar Bone Resorption

## Abstract

**Background:**

Periodontal disease is common in both developed and developing countries and affects around 20–50% of the global population, especially in adolescents, adults and the elderly is a public health problem. ADMSCs have the advantage of regenerating damaged tissue with high quality. DDM in the form of slices can improve healing in the mandibular sockets of molar teeth. The combination of ADMSC-DDM is expected to accelerate bone regeneration.

**Objectives:**

To analyze the combination of ADMSCs-DDM at increasing bone marker expression in periodontitis rats.

**Methods:**

This research is experimental with a randomized control group post-test-only design. A total of 50 male Wistar rats were divided into four groups: 1) normal group (K); 2) CP model (K + ); 3) CP model and treated with DDM scaffold therapy (K(s)); 4) CP model and treated with ADMSCs-DDM combination therapy (K(sc)). Making a CP model with injected LPS *P. gingivalis* into interproximal gingiva of the right first and second lower molars. The in vivo research stage was the implantation of the DDM scaffold and the ADMSCs-DDM combination in the rat periodontal pocket. Rats were euthanized on days 7, 14, and 28, and immunohistochemistry of STRO-1, RUNX-2, OSX, COL-I, and OCN was performed. DDM scaffolds are made in 10%, 50% and 100% concentrations for MTT testing. Statistical results were analyzed with Kruskal-Wallis and Mann-Whitney tests.

**Results:**

The results of the MTT scaffold DDM were significant in the 10%, 50%, and 100% dilution groups (p < 0.05). The results showed there was a substantial difference in the expression of STRO-1 between the study groups (p < 0.05). The (K(sc)) was significantly higher than the (K) in RUNX-2 expression (p < 0.05). OSX expression showed significant results between study groups (p < 0.05). The expression of OCN and COL-I showed a significant difference in all study groups on day 28, where the (K(sc)) was higher than the (K) (p < 0.05).

**Conclusions:**

Administration of the ADMSCs-DDM combination can accelerate alveolar bone regeneration on day 28. There is a mechanism of alveolar bone regeneration through the STRO-1, RUNX-2, OSX, and the COL-I pathway in periodontitis models.

## 1 Introduction

Periodontal disease is common in both developed and developing countries and affects around 20–50% of the global population. The high prevalence of periodontal disease in adolescents, adults and the elderly is a public health problem. Periodontitis etiology is a plaque and bacteria. Plaque bacteria secrete endotoxins which stimulate pro-inflammatory cytokines, resulting in the process of osteoclastogenesis. The process of osteoclastogenesis can cause alveolar bone resorption (Hernawati et al., 2023, Kitaura et al., 2020, Martin et al., 2021).

Mesenchymal Stem Cells (MSCs) possess a potency to improve the condition of several types of periodontal disease. They are multipotent, self-renewable, plastic, and able to multilineage-differentiating to osteoblasts, chondrocytes, and adipocytes. Mesenchymal Stem Cells have several advantages, such as regenerating damaged tissue with a high-quality result without fibrous tissue formation, minimal donor morbidity compared to autografts, and a low risk of rejection due to hypoimmunogenic responsiveness (Sanz,Antonio R;Carrion, Flavio S; Chaparro, 2015).

Adipose-Derived Mesenchymal Stem Cells (ADMSCs) are one of the sources of MSCs isolated from Adipose tissue, giving the advantage of easier access and sampling techniques that do not cause pain compared to bone marrow. Adipose tissue has the same characteristics as bone marrow in bone regeneration ability in vitro and in vivo studies (Dai et al., 2016, Lotfy et al., 2014).

ADMSCs have the advantage of regenerating damaged tissue with high quality, and high biocompatibility, and has the ability to self-renewal, plasticity, and multilineage differentiation. DDM has the ability of osteoinduction, and osteoconduction to stimulate new bone formation. DDM also serves as a scaffold for ADMSC to proliferate, differentiate to form a new network (New Bone).

Adipose-Derived Mesenchymal Stem Cells can also improve the healing of bone damage in the maxilla, mandible, and calvarium when combined with the scaffold. MSCs have high biocompatibility and can be combined with other bone material graf (Bassir et al., 2016, Martin et al., 2021). Recently, studies discovered that combining ADMSCs with inorganic bovine bones can stimulate the proliferation and differentiation towards osteogenic calvarial defects in mice with type 2 diabetes mellitus (Liang et al., 2013).

Demineralized Dentin Matrix (DDM) is a scaffold originating from bovine teeth; it is natural and has growth factors such as Bone Morphogenetic Proteins (BMP) and type I collagen (Reis-filho et al., 2012). Previous studies have suggested that autogenous DDM in the form of slices can improve healing in mandibular sockets of molar teeth. This shows that the dentin matrix possesses osteoinduction and osteoconduction capabilities and is a potent material graft (Hussain et al., 2012, Sudarmono et al., 2021).

Our study aimed to evaluate the reparative results of the ADMSCs-DDM combination in alveolar bone damage in rats. The combination of ADMSC-DDM is expected to accelerate bone regeneration.

## 2 Materials and methods

This research was conducted following the approval from the research ethics committee of the Faculty of Dentistry University of Jember No. 1623/UN25.8/KEPK/DL/2022. Our experimental procedure especially treated the rats already following ARRIVE and our animal ethical protocol institution.

### 2.1 Isolation and cell culture

Allogenic isolation of mice adipose tissue was obtained from inguinal fat by anaesthesia. Adipose tissues were washed with phosphate buffer saline containing 10% of antibiotic-streptomycin (Sigma-Aldrich, USA). Adipose tissues were diced with scissors and knives into small fragments, after which they were soaked in 0.2% type I collagenase (Worthington, USA) solution added with phosphate-buffered saline (PBS) and gently stirred for 40 min at 37 °C. Tissues were filtered using a 10 μm mesh filter (SPL, Korea) and were centrifuged at 1250 rpm for 5 min, and the supernatant was removed. MSCs were cultured with cell growth media (αMEM) (Gibco, USA) containing 15% Fetal Bovine Serum (FBS) (Biowest, USA) and 1% penicillin–streptomycin (Sigma-Aldrich, USA) and incubated at 37 °C with 5% CO^2^ levels. Cells were grown in 6 wells on plate tissue culture with a concentration of 100 in each well. After 3–4 times of passaging and reaching 80% confluence, we conducted the MSCs characterization by immunocytochemistry assay and flow cytometry.

### 2.2 Immunocytochemistry assay

The trypsinization step of single cells then followed by centrifugation procedure (125 rpm for 5 min). Pellets were added in stem cells medium, then embedded in glass objects, and incubated at 37 °C for 1 h. Fixation with formaldehyde (Bioworld, USA) was carried out, and washed PBS. Samples is added by anti-rat 105 FITC (Biolegend, USA), and anti-rat 45 FITC (Biolegend, USA), were incubated at 37 °C for 45 min. Furthermore, 50% glycerin is dropped on the object glass viewed with a fluorescent microscope; if the result is fluorescent, the results are positive.

### 2.3 Flow cytometry

The trephination step of single cells then followed by centrifugation procedure (125 rpm for 5 min). Pellets were added in stem cells medium. After that, those were fixed in a formaldehyde solution and closed BSA. Cells added by primary antibody CD105, CD 45 FITC (Biolegend, USA). Cells that have been fixed are analyzed by FACS Calibur flow cytometer.

### 2.4 Preparation of the demineralized dentin matrix from bovine teeth

The manufacture and processing of scaffolds from bovine teeth are carried out at the tissue bank of Dr Soetomo Hospital, Surabaya. The bovine teeth are extracted from the jaw with an osteotome, a hammer, and a saw. Teeth were cleaned with 3% peroxidase for 1 week. The teeth’ root, consisting of dentin and cementum tissue, is inserted into the bone miller and crushed so that it comes out in the form of particles—then filtered according to the desired size. The demineralization process using the bone mineral release method was added to 1% hydrochloric acid (HCL) for 1 day, washed clean, and dried. Before storage, the proteins contained in the teeth are frozen. Particles were made with sizes 355–710 µm and sterilized in BATAN (Jakarta, Indonesia). Analyzes of the bovine teeth particle shape was carried out by SEM with a magnification of 5000× (FK UA Electron Microscope Lab). The particles were also analyzed using micro-computed tomography (μ-CT) images (Lab μ-CT FMIPA ITB).

### 2.5 Scaffold seeding

DDM with 355–710 μm particle was immersed in αMEM medium for one day. After that, 5 mg of DDM was added to the 96-well culture plate, with ADMSCs. Then it was incubated for an hour at 37 °C and 5% CO2. The tubes are periodically shaken to distribute cells in suspension in DDM. DDM-ADMSCs were analyzed using SEM with 75 × and 1500 × magnification. (Electron Microscope Laboratory, Faculty of Medicine, Universitas Airlangga Surabaya, East Java, Indonesia).

### 2.6 Experimental animal groups allocation

This study used 50 male Wistar rats, 12–14 weeks old, with weight 200–250 g, with the following 4 group divisions:

1) The normal group (K), which did not undergo alveolar bone resorption, was not treated, and the subjects were sacrificed on the 14th day, consisting of 5 rats.
1) The control group (K + ), which was made alveolar bone resorption (Chronic Periodontitis (CP) model with Lipopolysaccharide (LPS) injection *P. gingivalis*), was not treated and sacrificed on the 7th (K(+)7), 14th (K(+)14), and 28th days (K(+)28), each consisting of 5 rats.
2) Periodontitis model rats treated with DDM scaffold therapy (K(s)), which was made alveolar bone resorption (CP) model with LPS injection *P. gingivalis*) was given DDM scaffold and sacrificed on the 7th (K(s)7), 14th (K(s)14), and 28th (K(s)28) days, each consisting of 5 rats.
3) Periodontitis model rats treated with ADMSCs-DDM combination therapy (K(sc)), which was made of alveolar bone resorption (CP model with *P. gingivalis* LPS injection) were given ADMSCs-DDM combination and sacrificed on the 7th (K(sc)7), 14th (K(sc)14), and 28th (K(sc)28) days, each consisting of 5 rats.

### 2.7 Periodontitis model

Before being induced by *P. gingivalis* LPS, the animal was anaesthetised using a combination of ketamine and xylazine. The dose given is ketamine 40–75 mg / kg and xylazine 5–10 mg / kg ratio 1: 1 intra-muscularly on the right posterior thigh with a duration of anaesthesia effect around 20–30 min. Experimental animals were previously inducted with *P. gingivalis* LPS (InvivoGen LPS-PG Ultrapure gene, Gen-San Diego USA) that was injected into their interproximal gingiva between the buccal aspect of the right first and second molars of the mandibula. Each experimental animal was given *P. gingivalis* LPS with a volume of 10 µl and a concentration of 0.5 mg / ml with a tuberculin one cc / ml syringe (Terumo) with a needle 30 G (BD), given three times a week for six weeks (Biomedical Laboratory, Faculty of Dentistry, University of Jember, East Java, Indonesia).

Periodontitis will show a decrease in alveolar bone with micro-computed tomography (µ-CT) with a parameter scan source voltage of 80 kV, a current source of 50 µA, an image pixel size of 24.2 µm, and exposure of 125 ms (Bruker Sky Scan 1173, High Energy Micro-CT, Faculty of Mathematics and Natural Science, Institut Teknologi Bandung, West Java, Indonesia).

### 2.8 Preparation of ADMSCs-DDM implantation in model periodontitis

ADMSCs cells were labelled with PKH 26. After being trepanned, ADMSCs cells were washed twice, resuspended with 1 mL, added, and routinely buffered according to factory rules. Suspension cells are mixed with the same volume of 1 mL liquid labelling solution containing PKH26 to reach the final concentration and labelled for 4 min at 25 °C, accompanied by periodic tapping with tubes. The reaction is rested with 1 mL of FBS added. Cells were washed twice in 5 mL αMEM. Using an MSCs fluorescent microscope labelled PKH26 will remove reddish fluorescents. DDM is placed in the periodontal pocket between the mandible’s first and second left molars, and the wound is closed with a periodontal pack. Group III was given ADMSCs cells labelled with 2 × 10^5^ cell / ml MSCs cell concentrations in 50 µl αMEM mixed with DDM, after which they were placed in a periodontal pocket of 0.1 cc between the first and second right mandibular molars. Furthermore, the wound closure with the periodontal pack. All animals experiment were not given immunosuppressive (Electron Microscope Laboratory, Faculty of Medicine, Universitas Airlangga Surabaya, East Java, Indonesia).

### 2.9 Immunohistochemical staining (IHC)

The rat was euthanized on the 7th, 14th^,^ and 28th days after implantation. Post euthanasia showed all the samples died, the right mandibular bone was taken. Mandibular bone, immersed in 10% buffered formalin fixation solution before decalcification and fixed in 4% PFA, dehydrated, embedded in paraffin, and cut into 3 μm sections. Parts are mounted on slides and processed for immunohistochemical staining. After deparaffinization and rehydration, the permeabilization portion with 0.4% Triton-X100 was blocked with 4% BSA and then incubated overnight at 4 °C with one of the following rats monoclonal primary antibodies: anti-STRO-1 (Abcam, USA), anti-RUNX2 (Abcam, USA), anti-osterix (Abcam, USA), anti-osteocalcin (Abcam, USA) and Collagen I (Abcam, USA). Preparations were measured in 10 fields of view with a light microscope with 400 × magnification.

### 2.10 Statistical analysis

Data are expressed as mean and SD. Statistical significance was determined using the Kruskal-Wallis and the Mann-Whitney tests to compare methods between groups, with p-values of<0.05 considered significant.

## 3 Results

### 3.1 Isolation and characteristics and flow cytometry of ADMSCs

**Fig. 1:**
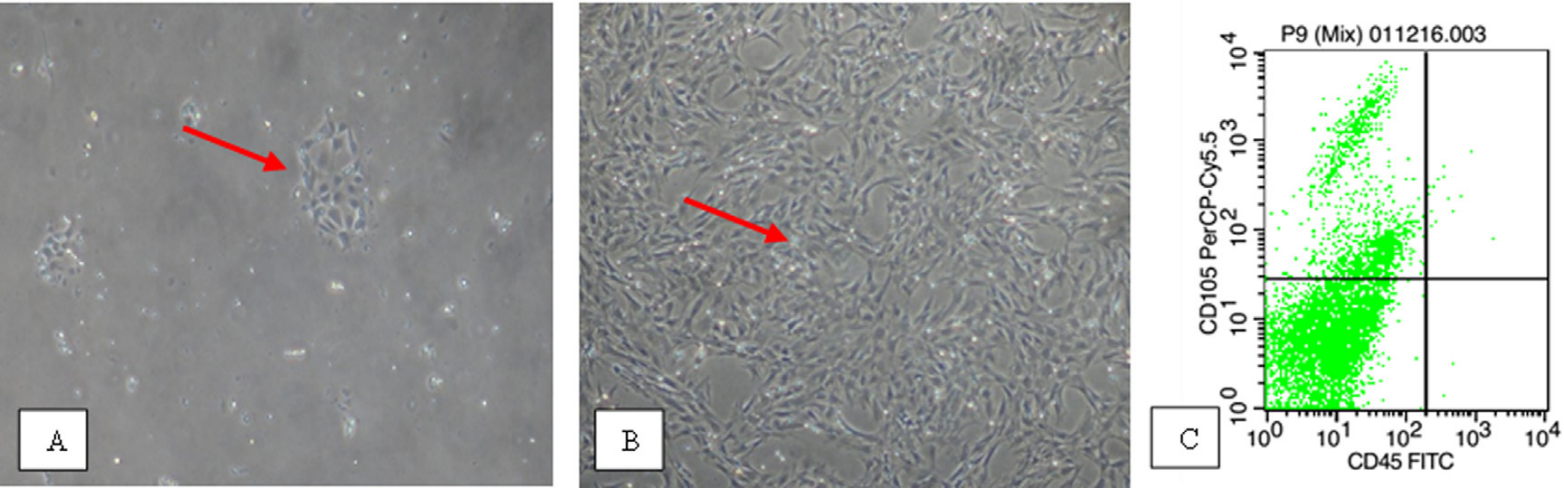
Morphological features of MSCs, which are shaped like fibroblasts and attached to the base plate; (A) Culture of MSCs in passage 1; (B) Culture of MSCs in passage 4 (Electron Microscope, 100 × ); (C) Flow cytometry shows that most of MSC’s subpopulations expressing CD105 are more dominant than those expressing CD45.

Observable progenitor cells were found from the isolation of ADMSCs on the first day, and after three days, when cell growth reached 80% of confluence, media replacement was performed. On the 15th day after passage 4, the cells filled the plate with fibroblast-like morphology (Fig. 1). The number of subpopulations of MSCs cells that express CD105 is more dominant at 77.67%, while the number of subpopulations of MSCs cells that express CD45 is only 22.07% (Fig. 1).

### 3.2 Osteogenic differentiation

Deposits of calcium and phosphate minerals cause cells to turn red after being cultured for 21 days in an osteogenic medium and stained with alizarin red. This confirms that MSCs cells from adipose tissue can differentiate in an osteogenic direction.

### 3.3 Scaffold DDM

**Fig. 2:**
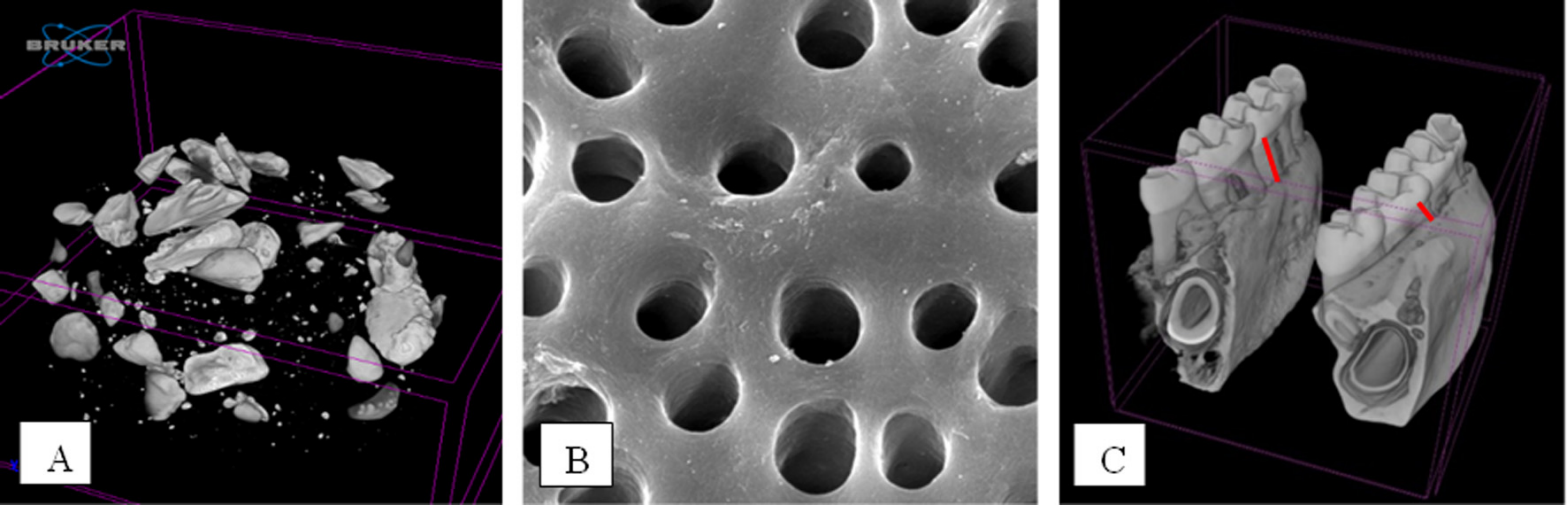
(A) Overview of the DDM scaffold particles (μ-CT photographs) (B) SEM photographs of DDM Scaffold visible pores (5000 × Magnification) (C) Overview of the periodontitis model shows alveolar bone resorption (red arrows indicate alveolar bone resorption (μ-CT photographs).

They are making DDM scaffolds at the Tissue Bank of Dr Soetomo Hospital Surabaya in particle form with a 355–710 µm diameter. The results with µ-CT showed that the DDM scaffold forms heterogeneous particles because of their differences in diameter (Fig. 2).

### 3.4 Seeding of ADMSCs-DDM

**Fig. 3:**
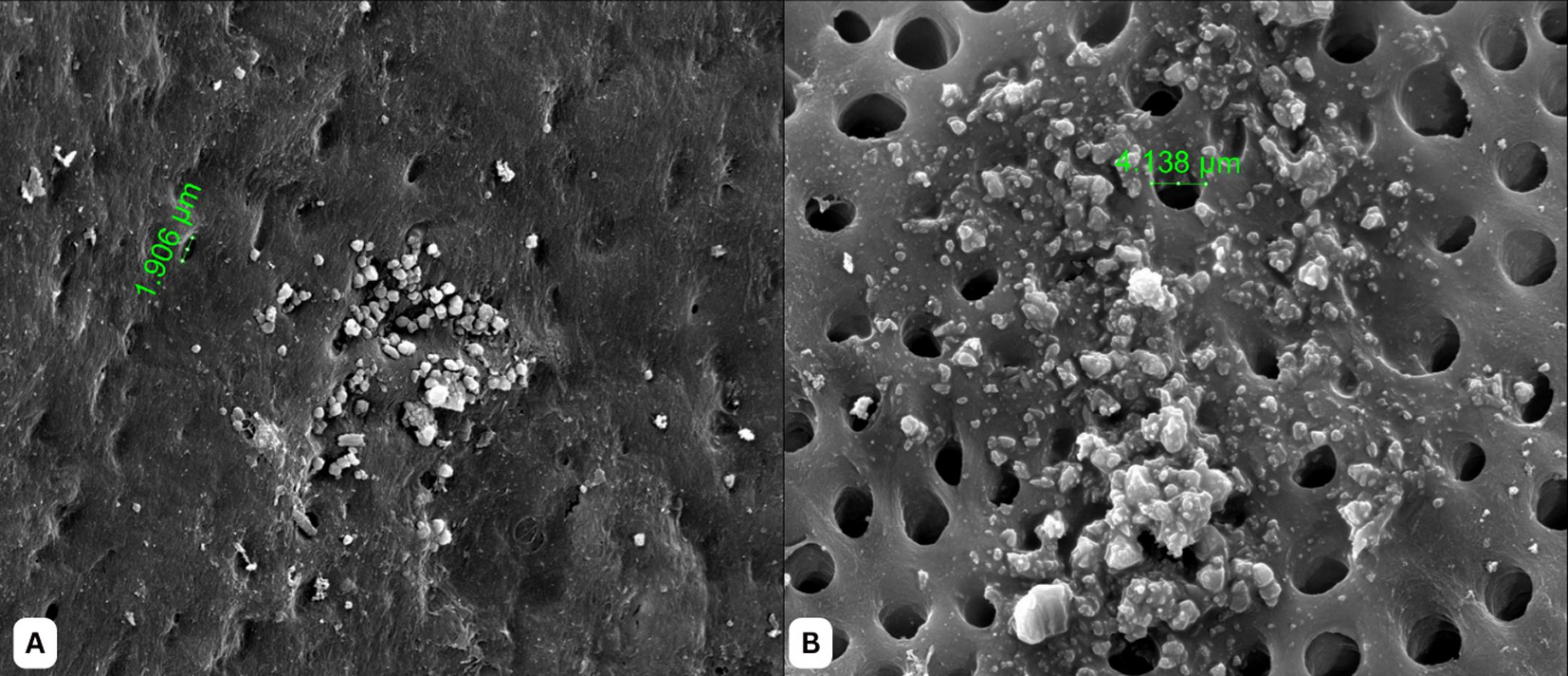
Seeding results of ADMSCs to DDM Scaffold using SEM (A) Seeding for 12 h (B) Seeding for 24 h (5000 × magnification, the red arrow shows the cell ADMSCs.

The seeding results of MSCs in 24-hour DDM scaffolds showed qualitative results; the quantity of MSCs cells in DDM scaffolds in the 24-hours group was greater than 12 h (Fig. 3).

### 3.5 Alveolar bone marker expression

**Fig. 4:**
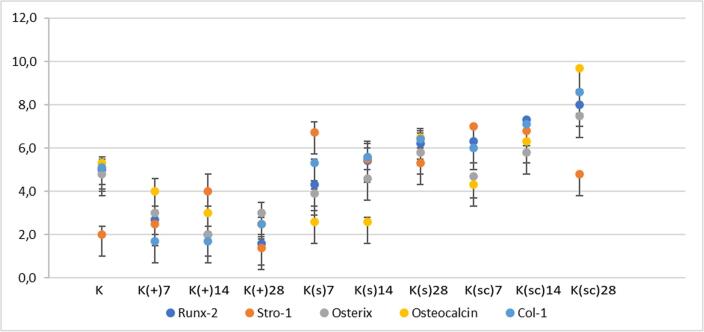
The result of alveolar bone biomarker expression on 50 samples of Wistar rats (Stro-1, Runx-2, Osteorix, Osteoccalcin, Col-1) used Kruskal-Wallis test with p-values of<0.05 considered significant.

**Fig. 5:**
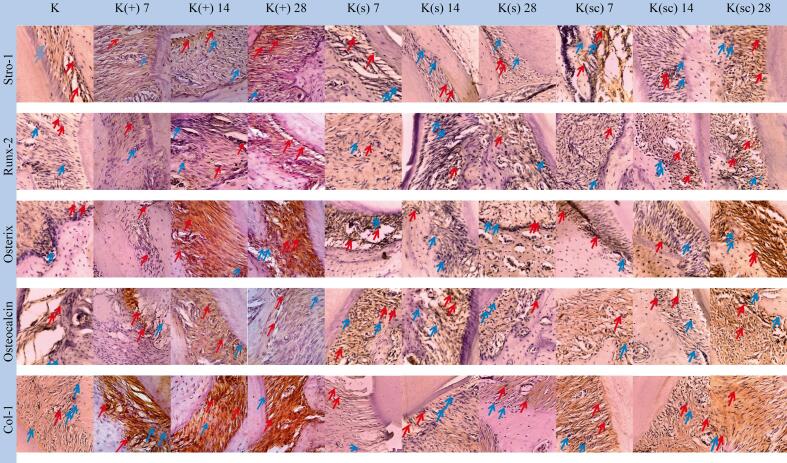
Representative histological images of alveolar bone stained with immunohistochemistry (magnification 400 × ), true positive expressions (red arrow), false positive expressions (blue arrow).

*Stromal expression of Precursor Antigen-1* (STRO-1) expressions in various groups can be seen in Fig. 4. There were significant differences between the study groups. Fig. 5 shows a significant difference in STRO-1 expression (p < 0.05) between the study groups. The administration of the K(sc) group was significantly lower than the treatment group, and the K(s) group (p = 0.000).

*Runt-related transcription factors-2* (RUNX-2) expressions in various groups can be seen in Fig. 4. There were significant differences between the study groups. The expression of RUNX-2 in various groups can be seen in Fig. 5 shows a significant difference in RUNX-2 expression (p < 0.05) between the study groups. The administration of the K(sc) group was significantly higher than the control group, the treatment group, and the K(s) group (p = 0.000).

*Osterix* (OSX) expressions in various groups can be seen in Fig. 4. There were significant differences between the study groups. The OSX expression in the various groups can be seen in Fig. 5. It shows a significant difference in OSX expression (p < 0.05) between the study groups. The administration of the K(sc) group was significantly higher than the control group, the treatment group, and the K(s) group (p = 0.000).

*Osteocalcin* (OCN) expressions in various groups can be seen in Fig. 4. There were significant differences between the study groups. The OCN expression in the various groups can be seen in Fig. 5. It shows a significant difference in OSX expression (p < 0.05) between the study groups. The administration of the K(sc) group was significantly higher than the control group, the treatment group, and the K(s) group (p = 0.000).

*Collagen type 1* (COL-1) expressions in various groups can be seen in Fig. 4. There were significant differences between the study groups. The COL-1 expression in the various groups can be seen in Fig. 5. It shows a significant difference in OSX expression (p < 0.05) between the study groups. The administration of the K(sc) group was significantly higher than the control group, the treatment group, and the K(s) group (p = 0.000).

## 4 Discussion

In this study, compared to DDM alone, the combination of ADMSCs-DDM showed faster new bone formation. On day 28, this combination showed active osteoinduction-deposition of alveolar bone on the surface of the bone matrix. (Mulyawan et al., 2022, Sari et al., 2019). The administration of allogenic MSCs might have a therapeutic effect on inflammation. A study by Eggenhofer et al., 2014 states that exogenous MSCs can stimulate endogenous MSCs to activate progenitor cells through the paracrine mechanism. (Eggenhofer et al., 2014) Exogenous mesenchymal stem cells are capable of inducing other stem cells in various body organs to move, restore damaged tissues, and regenerate the damaged alveolar bone. (Kawai et al., 2013, Pejcic et al., 2013).

The relationship between cells and scaffolds is where the scaffold releases a chemical messenger that binds to membrane receptors and affects intracellular communication. Growth factors bind to cell membrane receptors that initiate the intercellular cascade and affect gene expression; therefore, scaffold design is associated with osteogenic stem cell stimulation, which can eventually develop into a mature matrix of tissues and hard tissues (Shimauchi et al., 2013, Walmsley et al., 2016).

The tissue bank recommends a size of 250–750 µm for usage in dentistry. Previous studies stated that collagen combined with demineralized bone powder with a particle size of more than 250 µm is suitable for the environment of osteoblast differentiation and has the potential for tissue engineering. The combination with MSCs can accelerate and increase bone formation when compared to matrices alone (Koga et al., 2016, Polo-Corrales et al., 2014, Thitiset et al., 2013).

The result of this study indicates significant differences between experimental groups in the number of expressions of STRO-1, RUNX-2, OSX, COL-I, and OCN (p < 0.05). The K(sc) group presented in Table 1 shows the mean of STRO-1 decreased on days 7, 14, to day 28. In vitro, studies showed that STRO-1 expression increased on day 7 and decreased on day 42 in dexamethasone-stimulated hBMSCs cultures (Byers et al., 1999). The mean of RUNX-2 increased on day 7 and continued to increase from day 14 until day 28. The role of RUNX-2 has been proven in vitro, and several in vivo models reported that RUNX-2 expression peaked within 3–7 days in BM-MSC induced with the polyglycolic acid scaffold. The expression level was significantly higher than the control group after one week, which agrees with this study (Maher et al., 2015). The mean of OSX increased on day 7 and continued to increase from day 14 until day 28. In vivo study, implantation in rat muscle with a poly(lactide-co-glycolide) (PLGA) scaffold showed OSX plasmid DNA-induced mineralization after 28 days (Qiu et al., 2021). The mean of OCN increased on day 7 and continued to increase from day 14 until day 28. Osteocalcin is a specific gene secreted by osteoblasts. In an in vivo study, OCN expression was reported to increase on day 21 when mice had defects in the calvaria and were transplanted using nanofibrous fibrin scaffolds (Osathanon et al., 2009). The mean of COL-I increased on day 7 and continued to increase from day 14 until day 28. ALP expression with IHC showed higher on day 28 than in control (Tsai et al., 2011).

**Table 1:** Mean number and standard deviation of bone marker expression of experimental groups.

Bone Marker	Groups									
	K(-)	K(+)7	K(+)14	K(+)28	K(s)7	K(s)14	K(s)28	K(sc)7	K(sc)14	K(sc)28
Stro-1	2 ± 0,4	2,5 ± 0,6	4 ± 0,8	1,4 ± 0,4	6,7 ± 0,5	5,5 ± 0,5	5,3 ± 0,7	7 ± 0,2*	6,8 ± 0,3	4,8 ± 0,2
Runx-2	5 ± 0,5	2,7 ± 0,6	2 ± 0,4	1,6 ± 0,3	4,3 ± 0,4	5,4 ± 0,8	6,2 ± 0,5	6,3 ± 0,3	7,3 ± 0,3	8 ± 0,4*
Osterix	4,8 ± 0,2	3 ± 0,3	2 ± 0,1	3 ± 0,5	3,9 ± 0,2	4,6 ± 0,4	5,8 ± 0,2	4,7 ± 0,3	5,8 ± 0,6	7,5 ± 0,3*
Osteocalcin	5,3 ± 0,3	4 ± 0,6	3 ± 0,3	2,5 ± 0,3	2,6 ± 0,5	3 ± 0,2	6,5 ± 0,4	4,3 ± 0,3	6,3 ± 0,4	9,7 ± 0,6*
Col-1	5,1 ± 0,2	1,7 ± 0,3	2 ± 0,7	2,5 ± 0,3	5,3 ± 0,2	5,6 ± 0,7	6,4 ± 0,4	6 ± 0,2	7,1 ± 0,3	8,6 ± 0,2*

Group (K): normal group; group (K(+)7): control group, not treated and sacrificed on the 7th days; (K(+)14): control group, not treated and sacrificed on the 14th days; (K(+)28): control group, not treated and sacrificed on the 28th days; (K(s)7): periodontitis model rats treated with DDM scaffold therapy, was given DDM scaffold and sacrificed on the 7th days; (K(s)14): periodontitis model rats treated with DDM scaffold therapy, was given DDM scaffold and sacrificed on the 141 h days; (K(s)28): periodontitis model rats treated with DDM scaffold therapy, was given DDM scaffold and sacrificed on the 28th days; (K(sc)7): periodontitis model rats treated with ADMSCs-DDM combination therapy, were given ADMSCs-DDM combination and sacrificed on the 7th days; (K(sc)14): periodontitis model rats treated with ADMSCs-DDM combination therapy, were given ADMSCs-DDM combination and sacrificed on the 14th days; (K(sc)28): periodontitis model rats treated with ADMSCs-DDM combination therapy, were given ADMSCs-DDM combination and sacrificed on the 28th days. * There is a significant difference (P < 0.05).

The initial stage of STRO-1 indicates that MSCs have osteogenic precursors which can differentiate into osteoblasts. The process where MSCs differentiates into progenitor is the initial stage of the osteogenic process. In this process, RUNC-2 appears to increase on day 7 and decrease on day 28. The RUNX-2′s role was replaced by OSX. OSX increases in the final stage of the osteogenic process where osteoprogenitor cells differentiation on days 14–28, OCN and COL-1 were produced, which were the initial stages of bone mineralization (Di Tinco et al., 2020, Martin et al., 2021, Tsao et al., 2017).

Our results showed that ADMSCs-DDM combinations could increase type-I collagen expression on day 14 compared to day 7 (p < 0.05). Collagen type-I is an essential component of bone protein used for osteoblast differentiation. Stem cell interactions with COL-I via α2β1 integrins, which are the main signals for induction of osteoblast differentiation and matrix mineralization. Collagen type-I is also a marker of osteoblasts that are associated with cell proliferation and maturation (Jafary, F; Hanachi, P; Gorjipour, 2017).

ADMSCs-DDM combination can increase the area of alveolar bone trabeculae on day 14 compared to day 7. The process of bone remodelling is divided into several stages, where the 28th day is the stage of bone formation. At the bone formation stage, a new bone formation begins, and the early structure of COL-I expression starts at around day 14 (Hienz et al., 2015, Perez et al., 2015).

The reparative phase of the bone regeneration process occurs within two days to 2 weeks. During this phase, osteoblast cell proliferation occurs. This process is marked by the formation of a soft callus. These structures will then be replaced by fibrous tissue. The formation of the callus begins to experience ossification in the middle of the 14th day through the process of endochondral ossification. (Intini et al., 2014, Tobita et al., 2013) The switch of the osteoclast activity towards the osteoblast activity is called the reparative phase, which takes 7–14 days. Then the remodelling cycle draws preosteoblast from the bone marrow and forms mature osteoblasts, which synthesize bone matrix, especially COL-I, and regulate bone mineralization by increasing the width of bone trabeculae (Galler et al., 2010, Jayakumar and Di Silvio, 2010, Zhang et al., 2017).

Previous research stated that DDM is allogeneic for alveolar bone repair. DDM is one of the most promising biomaterials in dental tissue engineering because of its excellent ability to regenerate dental tissue DDM derived from human teeth is known to contain various biochemical factors such as BMP-2, Transforming Growth Factor-ß (TGF-ß), and basic Fibroblast Growth Factor (bFGF), while DDM derived from human teeth contains BMP-2, growth factor and non-collagen proteins. (Grawish et al., 2022, Han et al., 2021, Um et al., 2020).

This study did not make observations until the remodeling process was advanced and the results of the study only showed on the 7th, 14th, and 28th days so no represents the time stages of the overall regeneration process. This research was done within one month after the transplant so it cannot look at the development of cells, especially MSCs cells.

## 5 Conclusions

There are differences in the number of expressions of STRO-1, RUNX-2, OSX, COL-I, and OCN on the 7th, 14th, and 28th days after presenting the combination ADMSCs-DDM. Administration of the ADMSCs-DDM combination can accelerate alveolar bone regeneration on day 28. There is a mechanism of alveolar bone regeneration through the STRO-1, RUNX-2, OSX, OCN, and COL-I on the periodontitis model.

## Funding

Self-sustained.

## CRediT authorship contribution statement

**Desi Sandra Sari:** Conceptualization, Data curation, Writing – original draft. **Millenieo Martin:** Conceptualization, Data curation, Writing – review & editing. **Ernie Maduratna:** Investigation. **Hari Basuki Notobroto:** Formal analysis. **Ferdyansyah:** Methodology. **Ketut Sudiana:** Project administration. **Nora Ertanti:** Investigation. **Aristika Dinaryanti:** Investigation. **Fedik Abdul Rantam:** Supervision.

## Declaration of Competing Interest

The authors declare that they have no known competing financial interests or personal relationships that could have appeared to influence the work reported in this paper.
